# Academic expectations among international students from North-Western China: A case of technology use during and post COVID-19

**DOI:** 10.3389/fpsyg.2022.919702

**Published:** 2022-08-11

**Authors:** Abdo Hasan AL-Qadri, Salah A. M. Ahmed, Mohammed A. E. Suliman, Mohammad H. Al-khresheh, Azzeddine Boudouaia, Wei Zhao, Wenlan Zhang

**Affiliations:** ^1^School of Humanities and Education, Xi'an Eurasia University, Xi'an, China; ^2^School of Education, Shaanxi Normal University, Xi'an, China; ^3^Department of English Language, Faculty of Science and Arts, Northern Border University, Arar, Saudi Arabia; ^4^College of Education, Zhejiang University, Hangzhou, China

**Keywords:** COVID-19, academic expectations, international students, pandemic, lockdown, technology

## Abstract

This study examines the influence of the COVID-19 crisis on academic expectations among international students from north-western China. According to past studies, academic expectations are multifaceted, making it critical to test the methods employed to assess this fundamental trait. The outbreak of COVID-19 has resulted in various significant changes in education, which have shifted from traditional to online or mixed formats. As a result, examining international students' academic expectations along with their interactions with adopted technologies is a topic that addresses the current situation and issues. A mixed approach, comprising two different instruments (questionnaire and interview), was followed to achieve this primary objective. While a survey with a questionnaire was undertaken with 551 international students, divided into two groups, ten students were interviewed during and after the lockdowns. The findings revealed that COVID-19 had a significant impact on the academic expectations of students as well as many elements such as training for employment, personal and social development, international student mobility, motivation, social pressure, and social interaction with the help of supporting technologies. In terms of gender, men outperformed women in motivation, social interaction, training for employment, and personal and social development factors. Similarly, as per the grade variable (undergraduate, postgraduate, doctoral), the same higher trend was seen in postgraduates. Based on these findings, a set of recommendations was put forward. In the future, technology will be helpful in China's educational sector, such as online group collaboration, open education, managing student retention, and supervising teachers' recruitment.

## Introduction

When the novel coronavirus struck the world in the last weeks of 2019, no one thought that the virus would spiral into one of the worst pandemics of the 21st century (World Health Organization., 2020). Even after many countries announced preventive measures to keep the virus in check, no one in the academic fraternity foresaw that this would be one of the most challenging moments for the education sector (Al-khresheh, 2021). What started as a cautionary measure to check the virus soon became severe. Many colleges and universities took extreme steps, such as school closure with students being sent home as the virus surged worldwide. Although these measures were precautionary, the temporary campus closures of colleges and universities also saw enrollment dips and programs canceled, which mainly affected international students (Chen et al., 2020; World Health Organization., 2020).

A majority of international students studying in foreign nations felt the impact of the COVID-19 pandemic, with their study programs being thrown into jeopardy. In China, which has thousands of international students, the closure of all education centers brought fear, uncertainty, and anxiety. It took an emotional toll on these students who saw the virus as a disruptor of their degree programs and social welfare in a foreign land (Almroth et al., 2019).

The outbreak of COVID-19 happened at a time when the number of international students was increasing and studying abroad was gaining popularity. International students were forced to cut short the semesters they had spent years planning, and many of them were affected by the suspension of visa processing, while others saw their academic research funding being suspended. It became even worse for those international students planning to return to their respective campuses once the US government imposed travel restrictions. Besides, the disruption of international students' education calendar and the ensuing uncertainty over the coronavirus outbreak also affected individual students' academic careers (Braxton et al., 1995; Davis, 2006; Zivin et al., 2009).

Another expectation that international students have when they travel to foreign countries for further studies is to have social connections. The outbreak of the coronavirus and the introduction of preventive measures led to social and physical distancing measures that made it impossible to achieve that (Byrne and Flood, 2005). Besides, colleges and universities have the ideal and conducive environment to spread the virus given that educational institutions have several social spaces such as dining halls, dormitories, and theaters. Students usually congregate in these spaces in groups and in large numbers and could become potential hotspots for the outbreak of infectious diseases (Krammer et al., 2016).

International students were concerned that the virus outbreak would affect their studies and there were growing fears that the pandemic was disrupting their education and their livelihoods through travel restrictions and budget constraints. Since the outbreak of the pandemic, there have been restrictions on mobility, and the students studying in other countries have changed their study plans. While some considered enrolling in online courses or degrees, others opted to postpone their study programs altogether, which will see them stay longer before completing their respective classes (McCarthy and Kuh, 2006; Diniz et al., 2016).

The international students' expectations and plans were also put into disarray. They were worried about their financial positions, savings, and funding, which was likely to shrink due to the pandemic. Although the students studying abroad were more conscious of the online classes, the requirement that classes stop meeting in person and be conducted online with exams also done remotely proved costly to them due to financial constraints (Credé and Niehorster, 2011). For most students, finishing a college education is an important milestone. With many of them deferring programs, it was very discouraging as the COVID-19 pandemic was unlikely to disappear any time soon. For those students who hoped to get job placements and industrial attachments, the pandemic has been bad news as many job opportunities closed with governments requiring people to stay indoors to help check the virus spread.

The current study explores two theories: social cognitive career theory and self-determination theory. Social cognitive career theory “provides a framework to outline how individuals choose careers and maintain career fulfillment and stability” (Millán-Franco et al., 2019, p. 3). Qualities (e.g., self-efficacy, result aspirations, and individual goals), knowledge and socialization, and opportunities and obstacles all influence professional outcomes, according to the social cognitive career theory. Self-determination theory posits, “people are motivated to grow and change by three innate and universal psychological needs. This theory suggests that people can become self-determined when their needs for competence, connection, and autonomy are fulfilled” (Almroth et al., 2019, p. 788). Positive and realistic expectations reinforce motivation when faced with initial adaptation difficulties (Wang and Zhao, 2020). The findings of this study could help Chinese university international office staff build appropriate strategies and contribute to the study on the influence of COVID-19 on students' academic expectations. Furthermore, this research shows the important aspects influencing students' learning during and post-pandemic, as well as how to increase the AEs of students by using different technologies.

The current study included training for employment, personal and social development, international student mobility, motivation, social pressure, and social interaction as factors that were potentially influenced by COVID-19. Finding out how the pandemic shaped international students' academic expectations is essential in this context. This study addresses the main question: How are the academic expectations of international students in north-western China affected by COVID-19 during the pandemic as well as post-pandemic period, considering the educational system has changed from traditional to online learning mode?

The study also explored the following three sub-questions:

- Is there an acceptable validation of the current research questionnaire?
- What is the effect of gender on the academic expectations of international students?
- What is the impact of grades (level of education) on the academic expectations of international students.

## Literature review

Previous studies reveal that high academic expectations are linked to the most positive mental health outcomes. They may represent other unmeasured domains and variable processes such as shared educational values and positive prospects. According to the self-determination theory, external demands can also be internalized, resulting in intrinsic motivation (Almroth et al., 2019). Students at universities are at an increased risk of developing psychological symptoms (Zivin et al., 2009). Late adolescents struggle with loneliness throughout their transition to university. They are exposed to multiple stressors specific to this developmental period, suggesting that coping efficacy might have been a protective factor that contributed to daily physiological stress activity regulation (Drake et al., 2016) during the pandemic quarantine (Wang and Zhao, 2020).

According to social cognitive career theory, a career refers to the possible outcomes of pursuing a specific career path irrespective of whether the career brings results such as a pleasant environment and a good salary (Millán-Franco et al., 2019; Rasool et al., 2020). An individual's visualization of what will happen if they pursue a specific career route is defined by their result expectations. For instance, possibilities to serve others and develop a feeling of community may be included in the result goals for a job (Rasool et al., 2022; Zaman et al., 2022). According to social cognitive career theory, low career result expectations may discourage students from taking a given sort of career even if they have high self-efficacy (Millán-Franco et al., 2019). Based on item distribution, the academic expectations construct refers to training for employment, personal and social development, student mobility, motivation, social pressure, and social interaction.

Accordingly, with this multidimensional conception of AEs, Deaño et al. (2015), Diniz et al. (2016), and Almeida et al. (2018) found seven dimensions of expectations that students bring with them. Students have expectations about training for employment, personal and social development, student mobility, political/citizen involvement, social pressure, the quality of training, and social interaction.

### Training of employment

As one of the dimensions of academic expectation, training for employment (TE) is concerned with increasing the knowledge and skills of employees for doing specific jobs. In the context of students, it involves the growth of students' experience in all aspects related to the student's field of study (Diniz et al., 2016; Casanova et al., 2019; Alfonso et al., 2020). It can be related to training to gain significant employment or starting a career (Casanova et al., 2019). The seven dimensions of AEs identified by Deaño et al. (2015), Diniz et al. (2016), and Almeida et al. (2018) were obtained through multigroup confirmatory factor analysis (CFA). They seem to support a multifaceted and multidimensional concept across gender and nationality (Alfonso et al., 2020). While Casanova et al. (2019) investigated seven dimensions and removed one of them, the final questionnaire consisted of six dimensions after excluding the quality of training dimension. They found good validation of the remaining dimensions as employment training, personal and social development, student mobility, political enjoyment, citizenship, social pressure, and social interaction. The CFA confirmed all dimensions with a suitable fit index (Casanova et al., 2019). Also, expectations for service-learning varied with motivation, where those with high motivation also demonstrated higher expectations for engaging in the service-learning project regardless of their prior experiences (Muturi et al., 2013).

### Personal and social development

Personal and social development (PSD) is vital for students. It can be found in academic enablers and attitudes and behaviors that allow students to be successful in the school (Casanova et al., 2019). There are enough references and evidence that support the importance of students' personal and social development expectations. Personal and social development (PSD) encompasses freedom, self-assurance, reflective practice, and spiritual growth through a unique educational life (Casanova et al., 2019).

### International students' mobility

Student international mobility (SIM) is a standard key feature of knowledge transfer. More attention is paid to other features (Diniz et al., 2016; Casanova et al., 2019; Alfonso et al., 2020). It is related to the mindset of completing a portion of one's studies through overseas mobility programs, internships, or careers. When students' expectations and the academic reality they encounter are matched, they are more satisfied and more eager to graduate (Braxton et al., 1995; Davis, 2006; Casanova et al., 2019).

Positive and realistic expectations reinforce coping strategies when faced with initial adaptation difficulties (Nes et al., 2009; Krammer et al., 2016). Students are more likely to struggle to achieve their goals if their initial expectations are too high or unrealistic. Students become frustrated and engage less in learning new skills to cope with the demands of higher education in this situation (Byrne and Flood, 2005). Students enter college with different expectations (Schilling and Schilling, 1999; Krammer et al., 2016). Identifying these expectations helps teachers to adapt their classes to suit the needs of their students, such as assisting them in becoming intentional learners (McCarthy and Kuh, 2006; Diniz et al., 2016).

This attention is more critical when students enter university with different academic competencies and motivations, as well as various vocational or career ideas, or when they are first-generation students with a lack of sufficient and objective information concerning campus life (Braxton et al., 1995; Briggs et al., 2012; Alfonso et al., 2020). These students are often somewhat idealistic about what they can accomplish, and frustration can emerge in the first weeks of college life (Credé and Niehorster, 2011). The gap between initial expectations and subsequent experience can lead to dissatisfaction, disengagement, and poor academic performance (Banning, 2016).

### Motivation

At the same time, motivation (M) has been added in this study as one of the domains of academic expectations of students due to their attraction toward a particular task or objective that encourages them to search and engage in analysis to satisfy their interest within an established program (Muturi et al., 2013; Villegas et al., 2013; Almroth et al., 2019). Research on academic expectations points to its multidimensionality, aligning with the multiple experiences that students can have and develop in higher education (HE) (Casanova et al., 2019; Alfonso et al., 2020). AEs are defined as representations of what HE students expect to do during their academic life (Hassel and Ridout, 2017, p. 8) by interpreting their HE experiences in line with past experiences (Cole et al., 2009, p. 56). These AEs express HE experiences and academic knowledge (Banning, 2016, and confirmed by Soares et al., 2006), which, according to the interest and motivations of the students, is recoded and designed for new situations (Diniz et al., 2016; Alfonso et al., 2020).

### Social pressure

Social Pressure (SP) is a combined pressure that the students face in their everyday life, such as peer pressure, academic pressure, and socioeconomic pressure. These are the ones that students are familiar with. Individuals might think that they have complete control over these pressures, but their ideas may not be as clear as they are at another time when a situation arises (Seel, 2012; Diniz et al., 2016; Casanova et al., 2019; Alfonso et al., 2020). Social pressures can be confusing and stressful. It expresses an interest in participating in the state's political, cultural, and economic relations to help enhance it and join in specific associations or volunteer work. It covers issues relating to the desire to satisfy parental expectations or delights important others (Diniz et al., 2016; Alfonso et al., 2020). It involves a desire to have certain intervals of sociability and enjoyment, as well as a weekly period set out for these activities, separate from study hours, and which may require a connection with other students (McCarthy and Kuh, 2006; Diniz et al., 2016).

### Social interaction

The last domain included the academic expectations in this study is social interaction (SI). Previous studies have suggested considering it one of the essential academic expectations of students at the higher education level. Also, it is deemed a critical component of situated learning. According to Brown et al. (1989), learners are involved in a “community of practice” which embodies certain beliefs and behaviors to be acquired. They also argue that learning happens both inside and outside the classroom through collaborative social interaction and social construction of knowledge (Seel, 2012; Diniz et al., 2016; Casanova et al., 2019; Alfonso et al., 2020; Zaman et al., 2022).

The original academic expectations measure did not specify the six domains' contributions to the coronavirus situation. International students from different countries, gender, levels of higher education, and specializations were chosen for the study. To the best of our knowledge, this is the first study to assess the effect of the pandemic on the academic expectations of international students in China. In an unpredictable and evolving circumstance like COVID-19, it is natural to feel frustrated, nervous, or upset, among other feelings. Physical and psychological stress have been reported in medical personnel, children, patients with suspected infections, and quarantined family members (Cao et al., 2020; Chen et al., 2020; Wang et al., 2020). This study also contributes to a thorough understanding of the harm caused by COIVD-19, particularly its psychological effect on students regarding their education and ambitions (Cao et al., 2020; Wang and Zhao, 2020). Besides, investigating students' academic expectations (AEs) domains would facilitate researchers to understand better the effect of the COVID-19 crisis on AE outcomes among a group of international students who make up a significant portion of the student population in north-western China, where the current study was conducted.

### Use of supporting technology

The use of information and communications technology (ICT) during the COVID-19 pandemic witnessed different phases of integration with teaching and learning. Chinese professors, for example, used several free tools to present their courses online. Faculties discovered an opportunity to use virtual classrooms, virtual learning, and virtual teaching when they had to adapt to the constraints posed by the COVID-19 epidemic, and they used both synchronous and asynchronous methods. With the current economic crisis, technology is increasingly being used in the teaching and learning process. Higher education institutes in China have adopted online learning. During the lockdown, educators used Tencent Conference, WeChat (Luan et al., 2020), and Ding talk (Wiranota and Wijaya, 2021).

Tencent meeting or Tencent's VooV (https://voovmeeting.com/) could host 300 students, offered free services and connected individuals all across mainland China. In March 2020, its international edition, VooV Meeting, was introduced in over 100 countries and regions worldwide (www.tencent.com) to make it easier for people to access online learning and training (Lathifah, 2020; Wiranota and Wijaya, 2021). Tencent includes mini programs linked through WeChat as well as customized phone numbers and its sophisticated high-quality audio and video feature improved communication, meeting management, online document collaboration, real-time screen sharing, and instant text messaging to promote cooperation and teamwork during meetings (Sayibu et al., 2021; Wiranota and Wijaya, 2021). The application could also facilitate students to complete assignments, take examinations, and organize video conferences. Being recommended as an online learning platform (Wiranota and Wijaya, 2021), Tencent Meeting focuses on rapid communicative engagement between learners and instructors, which is electronically backed by cloud-based technology innovation for continuous education (Sayibu et al., 2021, p. 1). Use of ICT in education, therefore, could improve aptitude for lifelong learning, self-education, and information processing skills, make learning more active, engaging, and enjoyable, and increase interactivity and collaboration between teachers and students (Sadeghi, 2019).

### Conceptual framework

**Figure 1:**
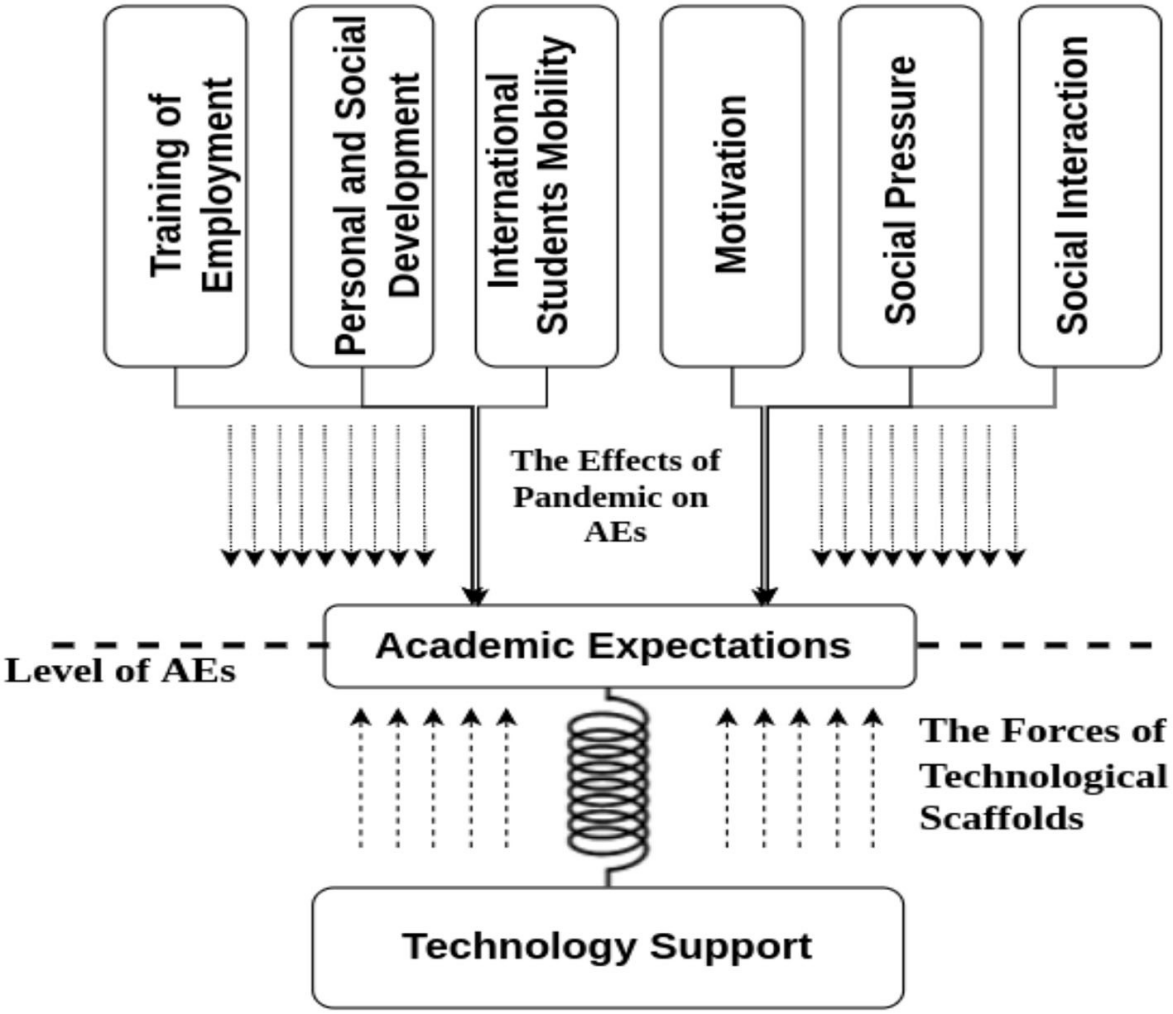
Conceptual model.

The current study is guided and informed by a theoretical framework, which also serves as a platform for future research. We employed the concept of technological scaffolding, which is demonstrated in Figure 1. Scaffolding is a method that helps students fulfill their learning objectives or complete a specific activity or task (Wood et al., 1976). It is one of the many characteristics of successful education that can be used in any setting. Although instructors may be enthused about scaffolding, it is essential to remember that scaffolding does not always mean teacher assistance. Scaffolding is a type of just-in-time support that provides students with the pedagogical nudge they need to work at a higher level. Various sociocultural scholars (Walqui, 2006) have established that learners are more likely to succeed when teachers and peers provide targeted help when required. Vygotsky claimed that socially situated learning, combined with scaffolding, resulted in better knowledge development and retrieval outcomes (Vygotsky, 1978; Dewey, 1986).

Thus, in the investigation of academic expectations of international students in China, most psychological aspects were investigated to determine how COVID-19 influenced them in China at various educational levels and ages. In addition, the current study examined the influence of supporting technologies as scaffolds that help maintain students' academic expectations under these critical conditions. The impact of the pandemic reduces academic expectations; thus, technological scaffolding is required to bolster the education process. Therefore, this theoretical foundation also guides technology integration under similar conditions. The reflection of students' AEs and the recognition of ICT's contribution based on this theoretical base has significance in various sciences and fields, such as educational, psychological, social sciences, and literature and research.

## Research method

### Research design

This research adopted an explanatory sequential mixed methods design that uses qualitative interviews to gain insight into international students' academic expectations. We used a two-phase (QUAN+QUAL) explanatory sequential design that started with the collection and analysis of quantitative data (questionnaire) that sought to compare variables after an action or event had already occurred (Salkind, 2010). This design was ideal for this study because it allowed us to collect data on the nature of students' academic expectations and the impact of the pandemic crisis on those expectations at universities in Xi'an city in north-western China. It helped us develop various approaches for students and professors to understand academic expectations during the teaching process.

The research tool (a part of the quantitative method) underwent three implementation phases. During the first phase, information about academic expectations instrument items was gathered and recorded. In the second phase, the opinions of academicians and experts regarding the face validity of the academic expectations instrument were collected. To determine the discriminant validity, average variance extracted (AVE), exploratory factor analysis (EFA), and CFA methods were adopted to investigate these constructs' validity. Additionally, Cronbach's Alpha, McDonald's Omega, and composite reliability were tested to serve the study's goals. In the third phase, the instrument was applied again by the same sample 'WeChat group when the epidemic crisis finished, and normalcy returned in China. Following the quantitative data collection and analysis, subsequent collection and analysis of qualitative data was undertaken (interviews) (Creswell and Plano Clark, 2011). The information gathered through an online survey and a semi-structured follow-up interview allowed for a more pragmatic and comprehensive understanding of the international students' expectations during and after the pandemic.

### Participants

International students enrolled in undergraduate and postgraduate degree programs on scholarships and self-financed at Chinese universities from 15 countries were randomly selected. They were from Pakistan, Yemen, Egypt, Tajikistan, Uzbek, Sudan, Ethiopia, Afghanistan, Iraq, Ecuador, Russia, South Africa, Japan, Vietnam, and Rwanda, and formed the population of the study. All students were non-Chinese and fluent in English.

The first samples comprised 551 undergraduate and postgraduate students from 30 different programs, such as human sciences, natural sciences, and applied sciences. Of the 551 students, 265 were male (48.1%) and 286 were female (51.9%) (Mean = 1.519, SD = 0.500); 249 (45.2%) were undergraduate students and 302 (54.8%) were postgraduate students (Mean =1.548, SD = 0.498). In their specialization, 208 (37.7%) students specialized in human sciences, 184 (33.4%) in natural sciences, and 159 (28.9%) in applied sciences (Mean = 1.911, SD = 0.812). The age of the students ranged between 18 and 40 years (Mean = 2.013, SD = 0.971). The methodology required the survey to be undertaken two times due to the nature of the study. The questionnaire was distributed to students staying in China at the beginning of the pandemic period. The second sample had 271 students and was used to calculate the impact of the COVID-19 crisis on the AEs of students at two time periods. The second application samples were lesser as some international students had left the country during the pandemic period. The second survey related to the time after students came back following normalcy returning to China.

### Instruments

Using the mixed approach, two different instruments were used in this study, a questionnaire and an interview.

#### Questionnaire

The academic expectations (AEs) scale of Casanova et al. (2019) was used in this study and adapted according to the theoretical base used in some studies. For instance, Diniz et al. (2016) and Alfonso et al. (2020), in their adaptation of the scale, used 24 items distributed along six domains: four items of training for employment adopted from Casanova et al. (2019), four items of personal and social development adopted from Casanova et al. (2019), four items of student mobility adopted from Casanova et al. (2019), four items of motivation adopted from Yi and Duval-Couetil (2018), four items of social pressure adopted from Casanova et al. (2019), and four items of social interaction adopted from Casanova et al. (2019). These were measured on a five-point Likert scale (Diniz et al., 2016; Casanova et al., 2019; Alfonso et al., 2020). In this study, motivation was added instead of political engagement citizenship due to the nature and goal of the study, which required measurement for motivation. The measurement domain was derived from Yi and Duval-Couetil (2018). Hence, it was shared with experts and specialists for their assessment and approval. The possible range of scores was 24 to120. The study tool was then tested on a pilot basis to support the panel's viewpoints and verify the preliminary psychometric properties (validity and reliability). The results showed the Cronbach's Alpha (α) = 0.723 and the square root of α that was used to determine the validity $=\sqrt{\alpha }$ = 0.850 (Smits et al., 2017; Al-Qadri et al., 2021), which indicated that reliability and validity values were acceptable (Heale and Twycross, 2015). This procedure was undertaken initially for supporting experts' and specialists' viewpoints.

#### Semi-structured interview

The interview protocol was designed to enable participants to explain their academic expectations during the COVID-19 pandemic. Table 1 illustrated the demographic information of the interviewed students. The interview included the same broad topic areas as the quantitative questionnaire (Diniz et al., 2016; Casanova et al., 2019; Alfonso et al., 2020). All interviews were voice recorded, followed the standard interview format, and transcribed and analyzed in the same manner. The interviews were conducted after two questionnaire applications to emphasize the effect and other identified hypotheses and differences in the content of the research instruments.

**Table 1:** Demographic information of the interviewed students.

**No**	**R-Name**	**Age**	**Gender**	**Nationality**	**Job**	**Major**	**Edu level**
P1	B.N	35	F	Vietnam	No	Media	PhD
P2	A.S	30	M	Japan	No	Engineering	Master
P3	F.M	20	M	Tajikistan	No	Chinese Language	Bachelor
P4	W.T	27	M	Afghanistan	Y	Medical	Master
P5	A.B	22	M	Uzbek	No	Economic	Bachelor
P6	S.L	27	F	Pakistan	No	Psychology	Bachelor
P7	M.F	32	M	Yemen	Y	Computer science	Master
P8	G.A	24	F	South Africa	No	pharmacy	Bachelor
P9	F.R	33	F	Egypt	Y	Math	PhD
P10	A.K	23	F	Rwanda	No	Microbiology	Bachelor

### Procedure

The questionnaire was written in English. It was then sent online *via* WeChat to all international students enrolled in undergraduate and postgraduate programs at different universities (Shaanxi Normal University, Xi'an Jiaotong University, Xidian University, Chang'an University, Northwestern Polytechnical University, and Shiyou University) in Xi'an city, China. The international students' office (ISO) and other students were additionally requested to share the links to the online questionnaire in students' groups. An informative message was sent to all students in the WeChat groups from February to April 2020 to participate in the study if desired.

A total of 551 undergraduate and postgraduate international students received the invitation to participate in the study. Only completely filled questionnaires were registered and included in the final sample to avoid missing values. Students had to read the information about the study and agree to participate to access the questionnaire. Only 271 students submitted their responses entirely as the second application was distributed to them by the same WeChat groups as some students left China at the height of COVID-19. The second application was in June 2020, after students returned to school (online and offline), to determine the degree of COVID-19 impact on academic expectations. Further, in addition to the iterated questionnaire survey, an interview with select students was undertaken to confirm the questionnaire results and provide a complete picture of the effect of COVID-19 on international students' academic expectations in Northwestern China.

### Data analysis

Varimax rotation was used in the analysis and main components analysis, including exploratory factor analysis (EFA), item analysis, and reliability analysis. Confirmatory factor analysis (CFA) was used to measure the suitability of the model to data in addition to determining factors underlying the definite measuring instrument (e.g., means, standard deviations, Cronbach's alpha coefficients, McDonald's Omega, CR, AVE, correlation matrix, *t*-test, KMO, Chi-Square, CFI, GFI, TLI, and RMSEA). Cohen's d found the effect size. Statistical analyses were processed by SPSS version 22 for descriptive statistics and EFA, AMOS version 24, and JASP were used for determining the fit indices and CFA.

Thematic analysis (Braun and Clarke, 2006) was applied to analyze the qualitative data. The study employed a deductive approach to finding the information supporting the quantitative study's findings, identifying themes based on semantic content. The second and third authors of this study familiarized themselves with the transcripts. Then they extracted descriptive phrases related to the participants' explanations of their responses to the questions in the quantitative questionnaire.

These phrases were discussed, and an agreement was reached between the second and the third authors. Then, the finalized codes and themes were extracted using the Nvivo11 qualitative data analysis package. Next, the authors independently reviewed the transcripts with their assigned codes and themes. Finally, the themes that support the quantitative finding are reported in this study.

## Results

### Quantitative data analysis and results

**Figure 2:**
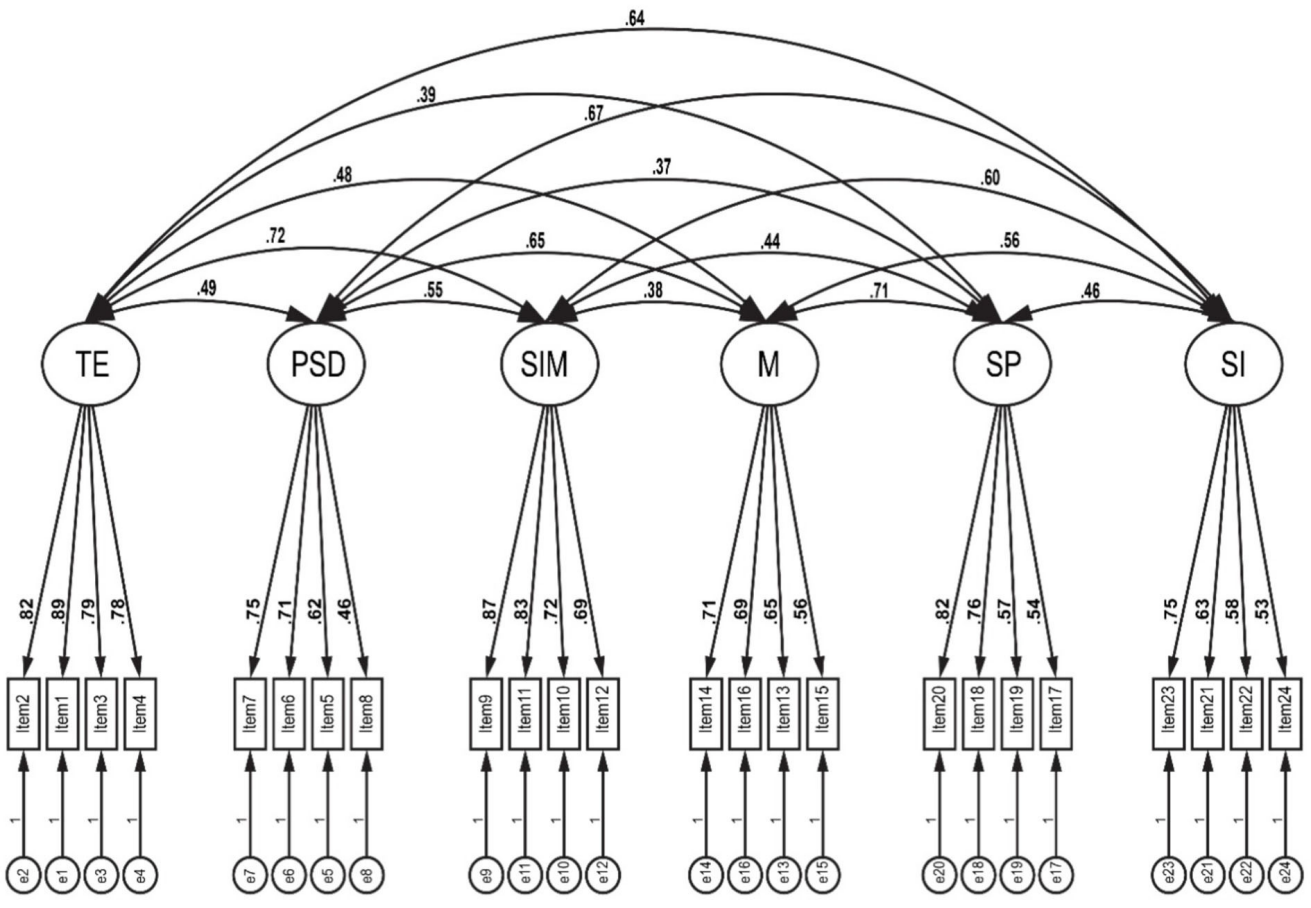
Measure structure model.

Researchers used the study hypotheses to examine and investigate the tool items that were confirmed and processed the study hypotheses outcomes. The principal component analysis examined the construct validity and determined the factors on which the items were loaded and for appropriate labeling of the factors. Kaiser-Meyer-Olkin (KMO) and Bartlett's Test of Sphericity (BST) were carried out to ascertain the appropriateness of the data for the analysis. The results displayed a KMO value of 0.771. Kaiser (1974) indicated that factor analysis should be carried out when the KMO value was more than 0.40 (Watkins, 2017), while Field (2009) implied KMO values above 0.60 to be acceptable. Table 2 illustrates the EFA, and all the AE tool items were loaded higher than 0.40 and considered good value (Blaikie, 2004; Al-Qadri et al., 2022). That suggested the factor item load should be at least 0.40. All the questionnaire items were confirmed through CFA (Figure 2). All items were higher than 0.50 in standardized loadings with keeping the same models. Values were acceptable, and the questionnaire was applied to the study population (Hair J. et al., 2014).

**Table 2:** Descriptive statistics and questionnaire validation.

**Variable**	**Item**	**Loading**	**M**	**SD**	**σ^2^**	**α**	**ω**	**CR**	**AVE**
Training for employment	2	0.964	3.818	1.082	1.171	0.713	0.745	0.801	0.695
	1	0.939	3.657	1.095	1.200				
	3	0.837	4.436	1.102	1.214				
	4	0.832	2.343	1.243	1.546				
Personal and social development	7	0.936	3.357	0.903	0.816	0.786	0.762	0.912	0.742
	6	0.835	4.096	1.093	1.196				
	5	0.823	3.877	1.350	1.825				
	8	0.805	2.735	1.178	1.388				
Student international mobility	9	0.909	3.697	1.018	1.037	0.855	0.813	0.931	0.771
	11	0.885	3.862	1.277	1.632				
	10	0.874	3.368	0.901	0.811				
	12	0.845	3.541	1.130	1.278				
Motivation	14	0.766	3.706	1.018	1.037	0.727	0.749	0.815	0.525
	16	0.761	3.702	1.024	1.049				
	13	0.701	3.724	1.019	1.040				
	15	0.664	3.535	1.261	1.591				
Social pressure	20	0.769	3.626	1.048	1.100	0.859	0.785	0.714	0.501
	18	0.647	3.624	1.071	1.148				
	19	0.612	3.617	1.053	1.109				
	17	0.432	4.045	0.793	0.629				
Social interaction	23	0.657	4.162	0.887	0.787	0.831	0.750	0.736	0.523
	21	0.539	3.499	1.153	1.330				
	22	0.515	3.417	0.863	0.745				
	24	0.485	3.379	0.890	0.792				

*M, mean; SD, Standard Deviation; σ^2^, Variance; α, Cronbach's Alpha; ω, McDonald's Omega; CR, Composite Reliability; AVE, Average Variance Extracted*.

The CFA was used to assess the measurement model in this six-factor model while keeping the same factor and items to emphasize the validity of the measured questionnaire. Chi-square value (10308.178; 0.001), root mean square error of approximation (RMSEA) = 0.067, comparative fit index (CFI) = 0.905, goodness of fit index (GFI) = 0.913, Tucker-Lewis index (TLI) = 0.904, and standardized root mean square residuals (SRMR) = 0.053 were all used to evaluate the measurement model. The guidelines for evaluating the adequacy of fit as proposed by Hair J. F. et al. (2014) are as follows: CFI and TLI values ≥0.90, RMSEA values with the upper bound at or less than 0.08, and SRMR values ≤ 0.06. All fit indices values were appropriate to use these instrument factor models for measuring the study aspects. As illustrated in Figure 2 below, all measures within each construct were loaded significantly on that construct. The final questionnaire consisted of 24 items within a six factor-model, with each factor having four items. The factors in the model were: (1) the training for employment model (2) the personal and social development model (3) the student mobility model (4) motivation model (5) social pressure model, and (6) social interaction model.

Given the above analysis, the value of Cronbach's Alpha was calculated based on the six-factor model for developing the study tool. The Cronbach's Alpha (α) for each factor was calculated as follows: the training for employment model = 0.713, personal and social development model = 0.786, student international mobility model = 0.855, motivation = 0.727, social pressure model = 0.859, and social interaction model = 0.831. Also, McDonald's Omega (ω) values were recorded, the training for employment model = 0.745, personal and social development model = 0.762, student international mobility model = 0.813, motivation model = 0.749, social pressure model = 0.785, and social interaction model = 0.750. For composite reliability (CR), the following scores were recorded: the training for employment model = 0.801, personal and social development model = 0.912, student international mobility model = 0.931, motivation model = 0.815, social pressure model = 0.714, and social interaction model = 0.736 as presented in Table 2. All the values mentioned above were suitable and acceptable ratios for this study's questionnaire (Deng and Chan, 2017; Watkins, 2017). These results are also in line with the findings of Tavakol and Dennick (2011). Consequently, the AVE was higher than 0.50 for each factor, indicating good convergent validity and referring to the study questionnaire's discriminant validity being acceptable (Heale and Twycross, 2015).

Group differences between male and female students can be explained as gender gaps in academic expectations. The question remains concerning how male and female students may have experienced the “effects” of various characteristics differently. To better understand these differential returns to their characteristics, we performed an additional analysis of the effect size on the academic expectation for each cohort by the gender variable. Researchers have already The coefficients for each domain between the male and female students in each cohort were compared using the *t*-test statistic recommended for this study sample, similar to those used in other educational research (Wells et al., 2013). Table 3 indicates the significant differences between male and female students. In TE and PSD, the values favor female students, while M and SI favor male students. However, there were no statistically significant differences between male and female students on SIM and SP.

**Table 3:** Effect of gender variable on the academic expectations of students.

**Domain**	**Gender**	**N**	**M**	**SD**	**t**	**df**	**P**	**CI 95%**		**Cohen's d**	**Effect- Size r**
								**Lower**	**Upper**		
TE	M	265	13.732	2.377	2.411^*^	549	0.016	0.815	0.083	0.206	0.102
	F	286	15.038	1.867							
PSD	M	265	13.758	2.654	2.049^*^	549	0.033	0.646	0.057	0.175	0.087
	F	286	15.035	2.328							
SIM	M	265	14.313	2.285	0.920	549	0.358	0.211	0.584	0.079	0.039
	F	286	14.612	2.429							
M	M	265	16.725	2.790	2.923^**^	549	0.006	0.971	0.042	0.249	0.124
	F	286	14.615	2.986							
SP	M	265	15.019	2.546	0.812	549	0.417	0.257	0.619	0.069	0.035
	F	286	14.815	2.656							
SI	M	265	15.991	2.384	1.987^*^	549	0.045	0.586	0.222	0.169	0.084
	F	286	14.812	2.412							

* *p < 0.05,*

** *p < 0.01.*

*TE, Training for Employment; PSD, Personal and Social Development; SIM, Student International Mobility; M, Motivation; SP, Social Pressure; SI, Social Interaction*.

The grade variable was examined and Table 4 details the differences. Training for employment (TE), personal and social development (PSD), motivation (M), and social interaction (SI) domains concerning grade variables (undergraduate and postgraduate) were significantly in favor of postgraduate students whereas no statistically significant difference was observed in international student mobility (SIM) and social pressure (SP) domains.

**Table 4:** Effect of grade variable on the academic expectations of students.

**Domain**	**Grade**	**N**	**M**	**SD**	**t**	**df**	**P**	**CI 95%**		**Cohen's d**	**Effect- Size r**
								**Lower**	**Upper**		
TE	undergraduate	249	14.008	2.189	5.544^***^	549	<0.001	1.362	0.049	0.473	0.230
	Postgraduate	302	16.457	2.164							
PSD	undergraduate	249	14.000	2.463	4.599^***^	549	<0.001	1.394	0.053	0.393	0.193
	Postgraduate	302	16.119	2.597							
SIM	undergraduate	249	14.570	2.331	1.44	549	0.139	0.894	0.107	0.127	0.063
	Postgraduate	302	14.384	2.391							
M	undergraduate	249	14.550	2.804	2.471^*^	549	0.038	1.346	0.064	0.211	0.105
	Postgraduate	302	15.765	2.643							
SP	undergraduate	249	15.012	2.434	0.90	549	0.358	0.732	0.140	0.079	0.039
	Postgraduate	302	14.831	2.737							
SI	undergraduate	249	14.357	2.519	2.57^*^	549	0.021	1.723	0.060	0.219	0.109
	Postgraduate	302	15.940	2.302							

* *p < 0.05,*

*** *p < 0.001. TE, Training for Employment; PSD, Personal and Social Development; SIM, Student International Mobility; M, Motivation; SP, Social Pressure; SI, Social Interaction*.

**Figure 3:**
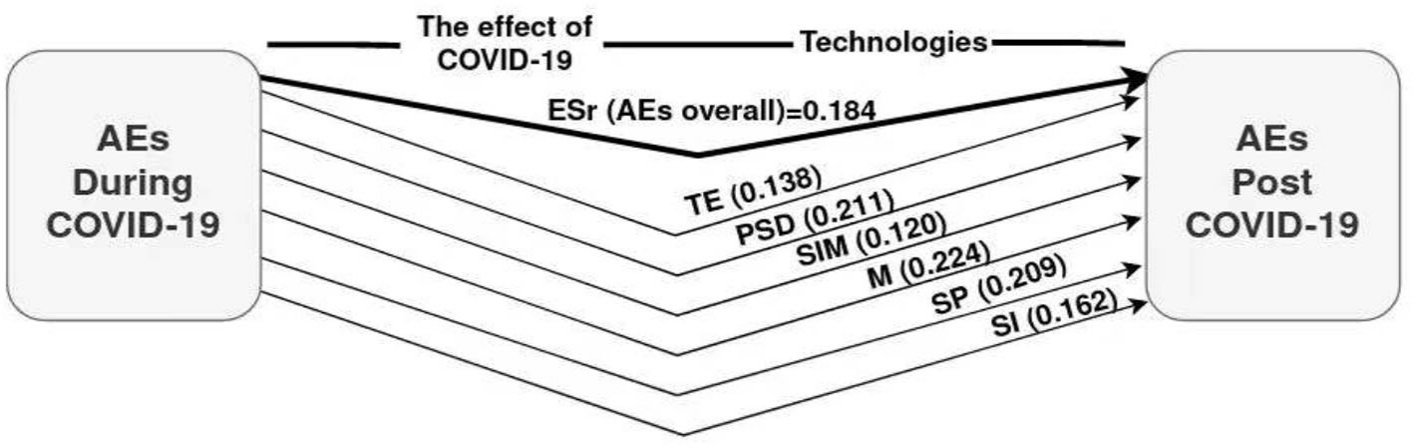
The effect size of COVID-19 on the AEs model.

The effect size of COVID-19 was identified by comparing the pre- and post-COVID-19 applications. However, the samples were different in the two applications. The pre- and post-COVID-19 comparisons were done with 271 samples (as only 271 were available in the post-pandemic count). A random selection of about 271 samples was taken from 551 pre-pandemic samples. There was no intervention as in the experimental studies, as only the influence of COVID-19 was assessed between two applications (suspension and return to school). According to the t-test, the effect size was determined by Cohen's d. Table 5 and Figure 3 explain the effect size of COVID-19 on students.

**Table 5:** T-test the effect of COVID-19 on the academic expectations of students (during –post) crisis.

**Domain**	**T-test**	**df**	**p**	**Cohen's d**	**Effect size r**
TE	2.280^**^	269	0.013	0.278	0.138
PSD	3.543^***^	269	<0.001	0.432	0.211
SIM	1.975^*^	269	0.047	0.241	0.120
M	3.768^***^	269	<0.001	0.459	0.224
SP	3.511^***^	269	<0.001	0.428	0.209
SI	2.689^**^	269	0.008	0.328	0.162
Overall	3.067^**^	269	0.002	0.374	0.184

* *p < 0.05,*

** *p < 0.01,*

*** *p < 0.001.*

*TE, Training for Employment; PSD, Personal and Social Development; SIM, Student International Mobility; M, Motivation; SP, Social Pressure; SI, Social Interaction*.

### Qualitative data analysis and results

Content analysis of the qualitative data revealed seven dominant themes that influence students' academic expectations: future job requirements, satisfaction with academic activities, quarantines, travel restrictions, development of self-discipline, relationships, social activity, and anxiety. Each is discussed in the following sections.

#### Future job requirements

The statistical results showed significant differences in training for employment between male and female students and students' educational levels. The qualitative data analysis emphasized the quantitative findings and revealed that all the interviewed participants had training goals to meet future career requirements. Most of the participants chose their majors based on the requirements of the workforce, and they expected to find jobs related to their current specialties.

“Because the major and the career had a special connection when I chose this major, of course in the future, I will have a job, something connected with my major”, she added. “Considering the changes that may occur in the labor market after COVID-19, but after I obtain my PhD, I will be fully qualified” (P1).

“My program is about China development and governance; it has a lot of effect on my future job because it studies how to manage and develop business and economy” (P4).

*Nevertheless, some expressed their concerns because the neture of their intended career or job changed post-COIVD-19:*

“I expect a good career after graduation after completing my master's, but I am afraid of many things like the situation under the COVID-19 pandemic. There are many things to reconsider” (P7).

Generally, international students have great expectations about the training at the Chinese host universities. The central focus was on mastering Chinese language skills. Most international students who study the Chinese language intend to use it in their future work.

“The biggest benefit actually that I can learn the language, I can master the Chinese language so, and it will be easy for me to find the job for example if I go to my country, I will be able to find a job with Chinese. Also, I can stay here to do business if I get a good recommendation from teachers” (P6):

*In another interview, a student stated:*

“China is one of the best countries, especially in business, and there are a lot of opportunities, so I am also looking for that. I expect to get the opportunity of finding a job or get some internship after the completion of the Chinese language program” (p7).

“The future job opportunity will be easily available because many Chinese companies and enterprises are opening in my country. That means it will be to my advantage that I can speak Chinese, which most of my people can't” (p8).

Another participant decided to find work different from his qualification because he studied philosophy and believed that it would be hard to find a job associated with that area.

“There are a lot of careers; considering my future career, I'm not relying on my master's degree. I am doing a master's degree in philosophy because I'm interested in philosophy, but I do not intend to find a job in the field of my master's degree in philosophy. I can do a job in a different field” (P2).

#### Satisfaction with the academic activities

Similar to TE, in PSD, the statistical results showed significant differences between male and female students and their educational levels.

*A South African student was not satisfied with her program; thus, she focused on learning the Chinese language:*

“The relationship between my future career and the area of my study, I feel like the relationship is not inclined because my program is different from what I'm pursuing now since I studied pharmacy in my bachelor but, I'm trying to focus on Chinese language and culture so, in the future, I hope it may add advantage to my previous degree certificate” (P8).

In addition, interviewed students expressed their plans for personal development, which they expected to obtain in the Chinese schools. A postgraduate student explained:

“I also got a chance to improve myself, my identity, and my personality because I think you face different people from you when you come to a different country. You got to learn something from them. I expected to get a chance to improve my self-confidence when I come so; this was my expectation” (P2).

Most of the students were aware of their own development needs, could recognize their weaknesses, and sought opportunities for self-development. However, they were not satisfied with their level of achievement and craved opportunities for self-development. The following excerpts clarify this:

“Uh, I am not satisfied because I have to learn a lot. I have to study more and work harder because everything will change, I have to learn more new things” (P4).

Another student expressed a higher expectation of his skills, but the current situation hindered his personal growth. “I think I have low achievements because of this situation”(P6).

#### Quarantines and travel restrictions

The measures taken due to COVID-19 had a great impact on the movement of foreign students, whether inside or outside China, and in this context, the Vietnamese doctoral student said:

“I want to go back home, but I could not because my flight was canceled…. and during this new wave, I think China is a safe place, so I am not getting back, for now, it is safe. I hope it will go like this way”(P10).

Everyone hoped that the epidemic would end and life will return to normal so that they could return to their countries or move around within China in search of work:

“I hope the Corona finish soon, so we can go to another city or another country, and get normal life so, I can get a job” (P9).

“If there is no Coronavirus, I think it will be easier to find a job than with corona. It is tough to find a job during coronavirus time. It affects China and the whole world” (P3).

“The thing right now I have some pressure under the COVID-19 pandemic I'm missing my home, I want to visit my home because I spent 1 year I did not back to my home” (P2).

Additionally, the pandemic's preventive measures have affected the students' ability to complete their research. For example, one of the students said:

“Just cannot go out to collect my documents or to collect information for my thesis” (P10).

Some students experienced panic and were worried about the future. One student thought that he would not be able to return to his home country due to travel restrictions and the increasing number of infections:

“I think I cannot go home…. sometimes I am terrified whether I will be able to go home, so this makes me feel sorry that I don't know what I can do” (P5).

#### Develop self-discipline

The interviews revealed the factors that support students' learning motivation, especially when the pandemic was at its peak. Most students were enrolled in postgraduate studies and were keen to obtain an academic degree. Thus, the international students shared how they developed a certain degree of self-discipline to complete their study requirements and graduate on time:

“I am really motived to complete my degree, but at some sort, I feel deep down in my heart I have to go home for once so, sometimes I say it is ok...I keep myself motivated due to the situation”(P2).

*An Egyptian student talked of her motivation regardless of the COVID pandemic*.

“I'm very much motived still obviously to process of the COVID-19 there was a time when lowered motivation but I guess I just had to convince myself and manage my time and everything just to be motived and complete my courses” (P9).

*Yet another student expressed worry over any potential delay in her graduation due to her age:*

“Because I am too old, I want to finish my degree quickly and get a job to earn money…Because of my age, I want to finish” (P1).

*A few others described their motivations during this challenging time and explained why their cause remained unaffected:*

“My motivation did not change as before; I have the same motivation; this pandemic cannot change my motivation because I get used to it” (P6).

“I have a great desire to pursue my high studies no matter how the situation is, and that this is the thing which makes me stay motivated “and “in these days we were studying courses normally in the classroom but, during the coronavirus, we desired to study, which made me keep self-disciplined during pandemic” (p7).

#### Social interaction and activities

To alleviate the pressure they faced, many students engaged in new activities or continued with earlier ones. These activities vary between individual or group activities. In this context, the doctoral student said:

“I do not want to get stressed, so; I already plant some trees in the school garden to keep myself busy with them; I just meet some neighbors in our dormitory” (P1).

“There are several social activities that m [sic] managed inside the campus like playing football and volleyball. I never played volleyball before, but during Covid19, my partners are both classmates and friends from my country” (P3).

*He also interacts with his friends, he added*.

“Yes, I also meet my friends, and going to friends and talking together” (P3).

*In contrast, an Uzbek student did not want to participate in any activity*,

“I have time for social activities, but I am an introverted person, so I am not attending any social activities. I feel stranger in such activities... sometimes I may go hang out with my friends just to have fun” (P5).

*Another student did not participate in any social activities; the student said:*

“We cannot do anything. We just stay in the room, nothing especial just cooking” (P9).

*Similarly, another interviewee shared:*

“I want to tell you the reality during the Covid19, I do not have social activities, just going to playground playing some sports like badminton or football like that… and staying in the dorm, not more than that” (P4).

*Another student, when asked to describe his social experience, stated:*

“You know it is pretty funny (laugh) because of the Covid19 there is no social life actually during Covid-19 pandemic, and also come together with my countrymen and speak together, I think if you stay alone, you will die (laugh) so social life was at the lowest level as it could be, no social life” (P10).

To show how ICT improved social activities and virtual communication, despite the physical distance, the African student said:

“We could not have social activities because during the pandemic I stayed in the dormitory, we were only quarantined, so the only social life activities I had were through social media on my phone or laptop…but I visit my friends who stayed in the dormitory with us, or we take a walk around the dormitory” (P8).

Additionally, to keep her mind off the current situation, she played a few games with other students, mostly volleyball or running around the school playground.

#### Relationships

Before the pandemic, the social activities were not only limited to their peers and international students reported that they had some social interaction and activities with their professors, such as gathering for dinners or lunches, or occasionally with staff members, and especially with their supervisors who always offered additional support for the international students. *Students described their relations with the professors and staff:*

“My relationship with them is very good they are really good they understand our situation, during pandemic they try to provide us with some help” (P3).

“Our relationship with the school members was really good during this pandemic every day in the morning and afternoon, and also at night they come to our room they ask us about our health situations, they take care of us too much because of that our relationship is better than before pandemic.” (P10).

#### Anxiety and social pressure

It is usual for international students in China to feel psychological pressure and anxiety due to the successive waves of the pandemic. This concern was not only self-concern but goes beyond that to include consideration for parents and relatives outside China with the pandemic reaching their countries of origin.

*The following quotes explain students' anxiety:*

“I experienced terrible feelings about my family, I was already stressed, but they thought I was safe in China” (P1).

“Currently, I am worried about them but not too much because some of my family members are doctors. They can take care of themselves”(P4).

*Another Japanese student clearly describes his fear:*

“Right now, I am apprehensive about them [his family] .... the infection cases are increasing day by day so, for them, it's necessary to go out. They cannot stay in quarantine for a week or a month because they have to do their jobs and don't have money to spend. It was a difficult time, I always told my family not to go outside the house, but as my mother is a doctor, she had to go. She is on the frontline, so I am afraid every day. I used to call my mother in the morning and evening; she used to it; this is her profession she has to do it”(P2).

An African student doing her bachelor's degree in pharmacy reported her anxiety about her parents “It is a little bit concerning because my father is a doctor constantly in contact with patients, but I keep peace of mind” (P8).

“I am very much motived still, obviously to process of the COVID-19 there was a time when motivation was lowered, but I guess I just had to convince myself manage my time, do everything, keep motived and complete my courses” (P9).

“It makes someone terrified; I feel uncomfortable and anxious when I heard about new waves of Corona pandemic, worrying about my family, my study and even myself” (P7).

In the beginning, many students were under pressure from their parents to return home. Some describe it by saying:

“My parents were concerned, so they kept calling me and asking questions using technological programs such as Wechat, which is commonly used in China and does not require a VPN. How are you? As any parents can do” (P3).

“They were worried about me every day they ask me every day they call me by supporting technology how about the situation? They were worried about me because they ordered me not to go out much every day during the first days of quarantine”(P4).

“At the beginning, they were trying to convince me to come back home, and they used to call me several times a day, and now it is the same as before” (P5).

Another student stated: “My parent, especially my mom, was worried about my well-being” (P10).

Fortunately, these fears began to ease with time, especially with the first wave of the pandemic subsiding, and many parents in their home countries learning how to deal with infected cases and protect themselves.

“My family members are very obeying rules and restrictions, and I was sure they would be quarantined at home” (P5).

*Another student described the pressure that resulted from the consequent measures of this pandemic:*

“Yes, the highest pressure, I do not know, especially the virus the virus covid19 like it is not used to be like before, different now and also the big pressure this year no much scholarship and the university is not providing scholarships because of the crisis, and also there is no motivation to study anything, this is the biggest pressure I think” (P6).

Students also faced pressure due to family expectations: “Yes, I found the program pressure higher than I expected and my family because I chose to do this program on my own without my family understanding the reason why I choose to do that, yes the pressure was a lot from my side. And it put setback on my personal need which is fine because I'm working on specific goal” (P8). Generally, the family's expectations were higher than the student's achievements.

## Discussion

This study's research findings on academic expectations confirmed the negative effect of the COVID-19 pandemic on international higher education in north-western China. The pandemic prompted most countries to opt for population social distancing measures to control the spread of the virus. Previous experiences have pointed out acute psychological effects (Odriozola-Gonzalez et al., 2020). The pandemic, similarly, has shown significant psychological symptoms related to anxiety, stress, and depression (Wang and Zhao, 2020).

This study explored the effect of COVID-19 on the academic expectations of undergraduate and postgraduate international students (who underwent quarantine in north-western China) during a pandemic through six domains derived from previous studies and provided to experts and specialists to investigate the validity of all instrument factors. The pilot testing applied and incorporated the experts' viewpoints on the instrument factors.

According to previous studies, factual validity was accounted for by EFA, and all items' loadings were higher than 0.40 and considered acceptable construction validity (Alfonso et al., 2020). The same instrument's items have been confirmed by CFA and matched previous studies relative to this measure (Diniz et al., 2016; Casanova et al., 2019; Alfonso et al., 2020). All items' loadings were 0.50 and above, as listed in Figure 2.

Cronbach's Alpha (α), McDonald's Omega (ω), and composite reliability (CR) attained high values indicating the tool was appropriate for this study (Heale and Twycross, 2015; Deng and Chan, 2017; Watkins, 2017)). The average variance extracted (AVE) values were higher than the correlation square values, indicating that discriminant validity had been investigated (Hair J. F. et al., 2014; Casanova et al., 2019). All values are listed in Table 2 and Figure 2. The first hypothesis regarding 24 items was distributed on the six factor-model of this study with acceptable validation (validity and reliability), and, therefore, was accepted.

Female students showed higher levels than male students in TE and PSD, whereas male students showed higher levels than female students in M and SI. However, no statistical differences appeared between the genders in SIM and SP. The results regarding TE and PSD are supported by a previous study on gender, planning, and academic expectations in first-year higher education students. Alfonso et al. (2020) reported that female students showed higher levels of academic expectations than males. The interviewees included both genders. This awareness was more evident in female students and consistent with the results of the questionnaire, as opposed to another study of first-year Spanish and Portuguese students (Diniz et al., 2016), with data collected at the beginning of the second semester; male students showed higher levels than female students in AEs (TE, PSD, SIM, and SP).

This study suggests that female students may have adjusted their initial expectations regarding TE and PSD. On the contrary, male students may interact and stimulate participation and contribution in their study life. Also, this study differs from some previous studies and established that there were no statistically significant differences between male and female students in SIM and SP. We believe that the reason for this could be that some demographic variables in this study were broader and covered two educational stages (undergraduate and postgraduate) to collect more details and suit the purpose of the study. Another study result (Diniz et al., 2016), found that gender directly affected five AEs. Similarly, in the present study, the direct effect of gender on TE, PSD, M, and SI was observed. However, it disagreed with the result by Alfonso et al. (2020), which found gender's immediate effect on two AEs (SIM and M).

The *t*-test was used to assess the role played by the grade variable on the AEs, in particular, to examine the direct effect and difference between undergraduate and postgraduate international students' AEs in north-western China. There were differences in training for employment (TE), personal and social development (PSD), motivation (M), and social interaction (SI) domains which were significantly in favor of postgraduate students. The outcomes from the interviews confirmed that it was very notable among doctoral students who had research commitments and professional dreams and felt the need to obtain an academic degree to search for a suitable job that matched their higher qualifications and expectations. No statistically significant differences were noticed in the international student mobility (SIM) and social pressure (SP) domains. It is apparent that students' grade level influences their academic expectations, and the higher the students' grade level, the higher their academic expectations were, as previous studies had reported (Hanover Research., 2012; Wells et al., 2013).

The literature reviewed in this study was used to test the effect of the COVID-19 crisis on AEs of the students, taking into account the pre-test (during) and post-test (after) COVID-19 crisis and return to school after the shutdown periods. The results of the tests (carried out two times) with the descriptive statistics are presented in Table 2. The study tool's process for the first application can be seen in Table 2 and Figure 2. The second application outcomes were compared with 271 respondents out of 551 respondents chosen in the first application. Measurement of the effect size was done by t-test for comparing AEs during and post-pandemic. Hence, Cohen's d was used to determine the effect size of COVID-19 on AEs domains of international students, as mentioned in Table 5.

The significance of COVID-19 on all the AEs domains were as follows: TE (T-test = 2.280; *P* ≤ 0.01; Cohen's d=0.278; ES=0. 138), PSD (T-test = 3.543; *P* ≤ 0.001; Cohen's d = 0.432; ES = 0.211), SIM (T-test = 1.975; P ≤ 0.05; Cohen's d= 0.241; ES =0. 120), M (T-test = 3.768; P ≤ 0.001; Cohen's d= 0.459; ES=0.224), SP (T-test = 3.511; *P* ≤ 0.001; Cohen's d = 0.428; ES=0.209), SI (T-test = 2.689; *P* ≤ 0.01; Cohen's d = 0.328; ES = 0.162), and overall (T-test = 3.067; *P* ≤ 0.01; Cohen's d = 0.374; ES = 0.184).

The statistically insignificant size can be attributed to ICT's role in reducing pandemic effects. It also refers to the basic difference between group means of the twice applications during and after the COVID-19 crisis. It supported these relationships, implying the negative effects of COVID-19 on AEs of students. Values referred to show the impact of the COVID-19 crisis on the AEs of international students. Despite this, the impact was small in some domains (TE, SIM, SI), and close to medium in other domains (PSD, M, SP). However, students were significantly influenced by all AEs domains during the COVID-19 crisis. Cohen's d was used to extract this type of effect size index, and classified effect sizes as small (d = 0.2), medium (d = 0.5), and large (d ≥ 0.8) according to Sullivan and Feinn (2012).

The interview outcomes emphasized the questionnaire outcomes, which were used as the first tool in this study. This was because the design of the interviews was consistent with the same areas of the questionnaire. The details of the qualitative analysis provided a profound insight that helped to understand the results of the quantitative data. It was noted that most students who enrolled in their majors hoped to obtain high-level training and qualification that met the requirements of the labor market, which is increasing day after day, especially considering the COVID-19 pandemic. Those who were interviewed provided details that substantiated their awareness of the changes in the labor market due to the pandemic.

Similarly, in terms of personal and social development, surveys and interviews revealed that participants were dissatisfied with the events and opportunities that allowed them to acquire specific social and professional abilities. This is because interviewees were more aware of these needs and provided more details than what was in the quantitative questionnaire. They clarified that they referred to exploiting opportunities, especially participating in online activities that promote their self-development and personality. With PSD, the students gained a better understanding of themselves and demonstrated patience until the situation was back to normal in China (Alfonso et al., 2020; Cao et al., 2020). This study's results are in line with the insights of previous studies on academic expectations (Villegas et al., 2013; Diniz et al., 2016) and other aspects of personal and social development needed for successful functioning in the school environment (Hassel and Ridout, 2017; Cao et al., 2020).

On students' mobility during the pandemic period, the analysis of the interview data revealed that everybody was affected by the preventive measures that restricted student movement. It affected the students' social lives and their ability to conduct research, thereby causing psychological damage and increasing anxiety levels among students of all educational levels.

Motivation (M) was the highest affected by the COVID-19 crisis. Expectations for service-learning varied with motivation (Muturi et al., 2013). The quantitative and qualitative results showed how students develop a certain level of motivation when affected by the situation, and the self-discipline to continue learning after a crisis was better than during it.

The data analysis provided an opportunity to identify the causes of the social and psychological pressures that students were exposed to during the pandemic. These went beyond the traditional reasons like the worry of failure or failure to achieve the expected success as a student or what their parents expected. Even students at higher educational levels experienced social and psychological pressures because of the COVID-19 crisis. This finding is similar to other studies that argued that increased anxiety and stress of students influenced SP (Alfonso et al., 2020; Wang and Zhao, 2020).

On social interaction, the results showed how students interacted with each other through social activities such as sports and trips organized by schools themselves. The findings also informed how these activities could help relieve anxiety and stress levels and help to promote healthy emotional development, especially under the pressure of the pandemic.

In general, it can be said that the impact of the COVID-19 crisis on SI, TE, and ISM, was negligible, as the beginning of the pandemic situation coincided with the Chinese autumn festival and the end of the first semester of the 2019–2020 academic year. Thus, the official vacation enabled students to trust the Chinese government to control the COVID-19 crisis while the International Student Office (ISO) at the Chinese universities and the student's supervisors provided daily health reports to each student to ensure the students did not lose their academic expectations (AEs). Moreover, learning resumed in March 2020, either through online study in some universities in an attempt to contain the situation and prevent the spreading of COVID-19 or face-to-face in others based on the nature of the situation in the province or city.

Our study also shows that the students from our sample were not highly influenced by the COVID-19 crisis and adjusted to the situation with a slight decrease in some AEs domains and close to medium in other domains during the COVID-19 crisis. The effect size criteria are listed in Kelley and Preacher (2012). More studies demonstrate the role of ICT and technologies in helping students reduce the psychological impact of the pandemic (Elhai et al., 2020; Browning et al., 2021).

The interviews also gave more details about relationships with teachers and faculty members and how male students, especially postgraduate, were more connected and involved, as they preferred to play some sports. In contrast, female students preferred individual activities, such as gardening or cooking, which are entirely consistent with the quantitative results. The recurring reason emerging through the qualitative results was anxiety due to the spread of virus infection. Students, regardless of gender and education level, explained how this anxiety increased over time on both sides (students and their parents). They were worried about each other and how it affected the students' academic expectations in light of an uncertain future caused by the pandemic. This study confirms the report of the Edge Foundation report on the educational and academic expectations of students and includes some of the research and perspectives put forward so far, bringing them all together in one place to comprehensively understand the impact of COIVD 19 on education, academic expectations, and youth labor market (Edge Foundation., 2020). Students have suffered a substantial psychological shock during the first weeks of the COVID-19 lockdown. This has influenced their academic expectations and other psychological aspects (Odriozola-Gonzalez et al., 2020; Wang and Zhao, 2020).

## Limitations and research directions

Due to the departure of most international students, the study samples of two applications (during and post the crisis) were not entirely matched. The number of post-graduate students in China during the pandemic was affected as long as the research focused on students living in China during and after the COVID-19 outbreak.

Furthermore, the study sample was selected from universities in Xi'an, and it can only be generalized to international students in Northwest China, and not all international students in China. Researchers did not include information about the country, scholarship type, participants' age, and whether the participants and or their family members were infected or not, all of which could be undertaken in future work.

Researchers only looked at the effect of COVID-19 on students' AEs in this study, not depression, post-traumatic stress disorder, or other psychological attributions, which will be further explored in future studies with innovation capabilities.

## Conclusion

According to the findings of this study, the COVID-19 pandemic had a moderate and significant impact on students' AEs. In line with the current study results, intervention programs to enhance academic success in HE should focus on planning at the beginning of studies, especially for men. Furthermore, according to the current research and the study of Diniz et al. (2016), such programs should also address gender differences, focusing on all AEs, especially Chinese women's AE. The AEs instrument for undergraduate and postgraduate international students in north-western China had sufficient validation and good fit indices. The tool was valid for measuring the effect of COVID-19 on students' AEs during and post the crisis.

This data may also be used to compare gender (male-female) and grade (undergraduate-postgraduate) differences, with statistically significant differences in employment training and personal and social development favoring the female category. Motivation and social interaction, on the other hand, supports men. However, there were no statistically significant differences between mobility and social pressure among international students regarding gender. In the grade variable, postgraduate students were significantly favored in employment training, personal and social development, motivation, and social interaction domains. At the same time, there were no statistically significant differences in the international students' mobility and social pressure domains.

The effect of the pandemic on international students who live in China was reduced by international students' offices in Chinese universities caring for and following students through regular health reports for each student *via* applications, adherence to lockdown, study shifts online, and wearing protective masks except when outside or in low-risk environments, and eating separately from each other for the students who live in China. In the future, some technological strategies will be helpful for China's educational sector, such as online group collaboration, open education, managing student retention, and supervising teachers' recruitment.

## Data availability statement

The raw data supporting the conclusions of this article will be made available by the authors, without undue reservation.

## Author contributions

AA-Q: collecting, analyzing, interpreting the data, reporting the draft, and proofreading the manuscript. SA: reporting the results and reporting the interview' outcomes. MS: recording the interviews, reviewing the literature, and proofreading the manuscript. MA-k: reviewing the literature and editing the manuscript. AB: interpreting the data and editing the manuscript. WeiZ: supervising the work and proofreading the manuscript. WenZ: supervising the work and checking the data and interpretation. All authors contributed to the article and approved the submitted version.

## Conflict of interest

The authors declare that the research was conducted in the absence of any commercial or financial relationships that could be construed as a potential conflict of interest.

## Publisher's note

All claims expressed in this article are solely those of the authors and do not necessarily represent those of their affiliated organizations, or those of the publisher, the editors and the reviewers. Any product that may be evaluated in this article, or claim that may be made by its manufacturer, is not guaranteed or endorsed by the publisher.
