# Supplementary material for: Academic expectations among international students from North-Western China: A case of technology use during and post COVID-19
Source: Front Psychol. 2022 Aug 11;13:919702. doi: 10.3389/fpsyg.2022.919702 (PMC9407684; doi:10.3389/fpsyg.2022.919702)
Supplement: Supplementary file 1 [file Data_Sheet_1.pdf]

Appendix (A): Questionnaire

Items	Questions	Source
**Training for employment**	1. Have better career opportunities in the labor market.<br>2. Get (online or offline) training to have a good job in the future.<br>3. Empower me to succeed professionally in the future.<br>4. Ensure a satisfactory professional career after the online course.	(Casanova, Almeida, Peixoto, Ribeiro, and Marôco, 2019)
**Personal and Social Development**	1. Take the academic opportunities to improve my identity, autonomy, self-confidence, etc.<br>2. Develop my personality.<br>3. Gain confidence in my potential, especially with the use of technology.<br>4. Acquire skills to be a more responsible and autonomous person, especially when attending online courses.	Casanova et al., 2019)
**Student international mobility**	1. Participate in university student mobility programs.<br>2. Arrange an internship in another country(via online mode).<br>3. Participate in student mobility, spending some time on online courses.<br>4. Be willing to take internships in another country to obtain an internationally recognized qualification.	Casanova et al., 2019)
**Motivation**	1. Focus on a technology that interests me<br>2. Satisfy a need in an online class<br>3. Solve a social problem<br>4. Create something of my own	(Yi and Duval-Couetil, 2018)
**Social Pressure**	1. Meet the expectations of my family.<br>2. Don't disappoint family or friends in terms of my academic achievement.<br>3. Fulfill the desire of those close to me who have encouraged my higher education.<br>4. Achieve a level of education similar or greater than that achieved by my parents (or older siblings).	Casanova et al., 2019)
**Social Interaction**	1. Have moments of socialization and fun with the use of social networks.<br>2. Participate regularly in virtual events and parties with colleagues.	Casanova et al., 2019)

	3. Have a group of friends with whom I can relax and socialize outside of class time.<br>4. Live and socialize with a new group of friends.	

Appendix (B): Semi-Structured Interview Protocol

Main Questions

Topic	Interview's questions
Background	Would you please tell me a little bit about yourself? age, academic background, job…etc.<br>• What is your social class, ethnic group?<br>• Do you have a job in your home country? What was your job?<br>• Do you have family, children? Their ages? Parents?<br>• How did they feel about you during the quarantine?<br>• How did you feel now about them during this new wave of Corona pandemic?
